# Editorial: Targeting autophagy in Cancer Therapy: Focus on small-molecule modulators and new strategies

**DOI:** 10.3389/fphar.2023.1145255

**Published:** 2023-02-01

**Authors:** Ryszard Pluta

**Affiliations:** Ecotech-Complex Analytical and Programme Centre for Advanced Environmentally-Friendly Technologies, University of Marie Curie-Skłodowska, Lublin, Poland

**Keywords:** cancer, autophagy, therapy, small-molecule modulators, new strategies

Cancers are one of the deadliest and most difficult to treat diseases in the world. The incidence of tumors in humans is constantly increasing and is one of the main challenges facing the health service. During the last 2 decades, remarkable advances have been made in diagnosing and developing new drugs to treat cancers through the use of genomics and high-throughput screening. Among them, autophagy plays an important role in tumors. Originally described as the phenomenon of removal and recovery of intracellular waste, autophagy has become a key biological process closely related to many types of cancer, suggesting that autophagy intervention is a promising therapeutic strategy in the development of anti-cancer drugs. Therefore, the discovery of cancer-related targets of autophagy and the related mechanisms of action of targeted small molecule drugs is of paramount importance. Autophagy plays a dynamic role in the inhibition or propagation of a tumor depending on its stage of development. In the early stages of tumor development, autophagy, as a survival and quality control phenomenon, prevents tumor formation. However, when cancers are at an advanced stage, autophagy, as a dynamic degradation and recycling system, contributes to the survival and growth of the tumors and promotes their spread through metastasis.

Although the pathology of cancer is fairly well understood, until recently we knew little about its molecular regulation. These mechanisms and their subsequent therapeutic orientation are the subject of our Research Topic. Of the 10 articles published on our Research Topic, four were original studies and six were reviews of currently known and emerging therapeutic targets for cancer treatment.

Thus, a high-quality database is crucial to elucidate the complicated relationship between autophagy and cancers, elucidate the crosstalk between key autophagy mechanisms and autophagy modulators with their remarkable anticancer activities. To achieve this goal, Fu et al. developed an extensive database of autophagic modulators that provided users with a high-quality practical online platform. Autophagic modulators database focuses on 153 tumor types, 1153 autophagic regulators including 903 activating, 191 inhibitory and 59 bidirectional compounds, 860 targets and 2046 signaling pathways (Fu et al.). Another original study by Yang et al. showed that cinaropicrin inhibited the growth of neuroblastoma cells *in vitro* and *in vivo*, through a mechanism involving endoplasmic reticulum stress/autophagy/NRf2 signaling/apoptosis. They provided insights into the mechanisms by which cynaropicrin induces apoptosis and endoplasmic reticulum stress-mediated protective autophagy (Yang et al.). This study indicated that cynaropicrin may be a potential antitumor agent for neuroblastoma prevention and treatment (Yang et al.). This study provided a basis for future preclinical and clinical trial exploration to improve the efficacy of cynaropicrin in neuroblastoma treatment. Chen et al. study presented that nuclear division cycle 80 might be a diagnostic and prognostic biomarker in lung cancer. Furthermore, elevated nuclear division cycle 80 expressions were detected in ionizing radiation resistant no small lung cancer cells, and was found to induce radiation resistance (Chen et al.). These findings provide novel insights into the effect of nuclear division cycle 80 on radioresistance in cancer cells, and suggest that nuclear division cycle 80 could serve as a drug target for improving radiosensitivity (Chen et al.). Last original work by Shi et al. discovered by *in silico* and *in vitro* screening four compounds 8012-0567, 8018-6529, 8018-7168 and 8018-7603 with inhibitory activities target PARP-1. Further cell assays showed that compounds 8018-6529 and 8018-7168 could inhibit the growth of the human colorectal cancer cell with an induced autophagy process and provide potential hit compounds for the development anti-cancer drug (Shi et al.).

Therefore, the autophagy pathway is highly drug-sensitive and has multiple drug targets. The inhibition of cytoprotective autophagy in the treatment of cancer is gaining importance as a potentially new therapeutic approach in the treatment of cancer (Zhang et al.). From a clinical perspective, new inhibitors targeting autophagy modulation are currently under investigation and small molecule inhibitors continue to show promise in cancer treatment (Zhang et al.). Review by Wei et al. summarizes the experimental validation and practical application of the strategies using specific and dual inhibitors, drug combinations and antibody-drug conjugates, with the intention of aiding and inspiring future research on nicotinamide phosphoribosyltransferase-targeted oncology drugs. The further development and design of novel, selective, highly efficient, low toxicity and low molecular weight nicotinamide phosphoribosyltransferase inhibitors will not only help basic pathology study, but also bring great hope for the clinical therapy of tumors involving nicotinamide phosphoribosyltransferase to the benefit of more patients (Wei et al.). A promising cancer treatment, which combines sonodynamic therapy with autophagy inhibition using a nanoparticle delivering system, is presented by Zhang et al. Authors speculated that the combination of targeted delivery of sonosensitizer and inhibition of autophagy can effectively kill cancer cells and also avoid the activation of protective-autophagy by increasing the production of reactive oxygen species (Zhang et al.). Autophagy connected genes, miRNAs, lncRNAs, and circRNAs have been described as autophagy biomarkers. As well, developing specially designed drugs for the abovementioned autophagy-associated biomarkers by high-throughput screening, molecular docking, and molecular dynamic simulation may be promising in improving oral squamous cell carcinoma therapy (Gou et al.). Qiang et al. presented that modulating autophagy has been emerging as a promising strategy for colorectal tumor therapy, which can benefit the patients who are not suitable for traditional treatment, and can be used as adjuvant, polypeptides, and small-molecule compounds, photodynamic and non-coding RNAs chemotherapies to overcome drug resistance. New experimental facts, from *in vitro* and *in vivo* investigations, suggest a context-dependent anticancer manifestation of autophagy, mediated by an injurious consequence on cancer stem cell survival and metastasis (Troumpoukis et al.). Even though autophagy is a process opposite to apoptosis, evident autophagic influx can in fact trigger apoptosis under certain conditions by activation of caspase eight and the reduction of endogenous apoptosis inhibitors (Troumpoukis et al.).

Taken together, the identification of new drug targets and the development of new treatment strategies based on them offer the exciting prospect of more effective therapies in the treatment of cancers. I thank the authors for their contributions and hope that each article on this Research Topic will both inform and generate further interest in the effective treatment of tumors.

